# Adhesion Behavior in Fish: From Structures to Applications

**DOI:** 10.3390/biomimetics8070534

**Published:** 2023-11-10

**Authors:** Jinhao Wang, Shukun Wang, Long Zheng, Luquan Ren

**Affiliations:** 1 Key Laboratory of Bionic Engineering, Ministry of Education, Jilin University, Changchun 130022, China; wangjinhao0533@163.com (J.W.); lqren@jlu.edu.cn (L.R.); 2 School of Mechanical and Electrical Engineering, Changchun University of Science and Technology, Changchun 130022, China; wsk@cust.edu.cn; 3 Weihai Institute for Bionics, Jilin University, Weihai 264402, China

**Keywords:** adherent fish, classification, adhesion mechanisms, underwater systems, bionic applications

## Abstract

In nature, some fish can adhere tightly to the surface of stones, aquatic plants, and even other fish bodies. This adhesion behavior allows these fish to fix, eat, hide, and migrate in complex and variable aquatic environments. The adhesion function is realized by the special mouth and sucker tissue of fish. Inspired by adhesion fish, extensive research has recently been carried out. Therefore, this paper presents a brief overview to better explore underwater adhesion mechanisms and provide bionic applications. Firstly, the adhesion organs and structures of biological prototypes (e.g., clingfish, remora, *Garra*, suckermouth catfish, hill stream loach, and goby) are presented separately, and the underwater adhesion mechanisms are analyzed. Then, based on bionics, it is explained that the adhesion structures and components are designed and created for applications (e.g., flexible gripping adhesive discs and adhesive motion devices). Furthermore, we offer our perspectives on the limitations and future directions.

## 1. Introduction

Through hundreds of millions of years of competition and survival, fish, the most common organisms in the ocean, have developed physiological structures and behavioral mechanisms that are extremely well-adapted to their environment. When it comes to fish bionics, people always think of mimicking the efficient, high-speed, and highly maneuverable swimming style of fish. This is because researchers have demonstrated that the fish swimming style outperforms the traditional propeller propulsion used for ships or underwater vehicles in terms of propulsion efficiency, speed, acceleration, maneuverability, and stealth [1]. Therefore, existing reviews in the literature are mainly focused on the swimming pattern and propulsion mechanism of fish. However, not all fish rely on this swimming pattern and propulsion mechanism. Most benthic fish that live in bottom waters have adopted a unique pattern of survival, evolving highly developed adhesive organs that can attach to various objects underwater. This behavior not only allows them to feed but also helps them to avoid predators and to move flexibly through a variety of small and complex underwater environments. In addition to this, the greatest advantage of this type of swimming is the reduction in energy expenditure associated with movement.

In order to adapt to the living environment of the bottom waters, these fish gradually possessed biological adhesive discs that evolved from different organs. The shapes, structures, and adhesion mechanisms of fish adhesive discs also vary depending on the application situation. For example, clingfish living on rocky intertidal zones have evolved ventral adhesive discs to resist the impact of surges, which allow them to perform rapid, reversible adhesion activities and even launch predatory attacks on attached molluscs [2,3]. Remoras hitch a ride on various hosts with the help of adhesive discs evolved from their dorsal fins, not only saving energy but also obtaining free food [4,5,6]. In addition, the suckermouth catfish and *Garra*, which inhabit the bottom of freshwater, are able to use their sucker-like mouthparts to create negative pressure for underwater adhesion, along with the tooth-like or spine-like unculi around their lips to scrape food from rocks [7,8,9,10,11,12,13]. Gobies and hill stream loaches can even use their highly evolved pectoral fins to achieve adhesive crawling, adapting to fast-flowing environments while facilitating rapid feed or escape [14,15,16,17,18,19,20,21,22]. Above all, understanding these properties and principles of animals, sometimes referred to as ‘biological understanding’ [23], will provide important insights into biology and engineering.

Bionics is a comprehensive science that imitates biological principles to construct technology systems or makes artificial technology systems that have the characteristics of biology [24]. The emergence of bionics opened an epoch in which humans moved from “taking from nature” to “learning from nature” [24,25,26]. In recent years, there has been a growing demand and interest in underwater actuators or systems to eliminate the risk of human involvement in ocean exploration. Learning from adherent fish can solve precisely many of the application challenges of underwater systems. For instance, underwater flexible gripping discs that mimic clingfish or remoras eliminate the need for complex power units and are capable of gripping irregularly shaped objects [27,28,29,30,31,32]. Underwater vehicles can also mimic the movement patterns and anchoring methods of benthic fish to extend their operating time and reduce energy consumption [33,34,35,36,37,38,39]. Therefore, people can apply the idea of bionics to more underwater systems by studying and learning the unique adhesive disc structure, adhesion mechanism, and even the movement pattern of adherent fish. This will play an important role in areas such as resource exploration, biological observation, military activities, and even medical health [40,41,42,43,44,45].

This review is organized as follows. Section 2 is divided into three subsections based on the different organs of adhesion in fish: those that use highly evolved fins for adhesion, those that use sucker-like mouthparts for adhesion, and those that do so with the coordination of the two. Section 3 describes the existing non-bioinspired and bioinspired adhesion systems. Section 4 gives the constraints, limitations, and future recommendations for existing bioinspired underwater adhesion systems. Section 5 completes this review with final conclusions and a final analysis of the gathered information.

## 2. Adherent Fish

**Figure 1:**
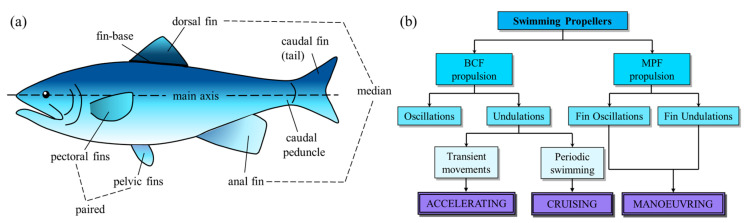
Terminology in fish propulsion patterns and the relationship between propulsion patterns and swimming functions （Adapted with permission from Ref. [47,48]. Copyright 2017, Elsevier Ltd., Copyright 1999, IEEE). (**a**) Terminology to identify the fins and other features of fish. (**b**) Diagram showing the relation between swimming propellers and swimming functions.

Traditionally, researchers working in the field of biomimetics adopt the nomenclature introduced by Breder [46] to classify the swimming modes used by fish: body and/or caudal fin (BCF) propulsion patterns and median and paired fin (MPF) propulsion patterns. The terminologies used in the literature are described based on the morphological characteristics of fish shown in Figure 1a. BCF and MPF thrust-generation mechanisms are further classified as undulatory or oscillatory [47,48,49,50,51,52], and correspond to three basic optimal designs for accelerating, cruising, and maneuvering [53] (Figure 1b).

**Figure 2:**
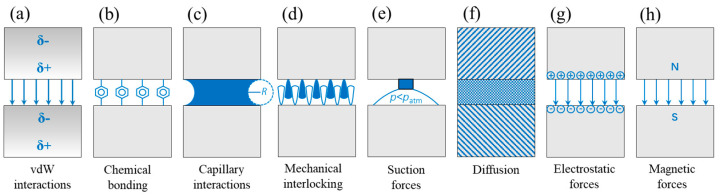
Different adhesion mechanisms resisting the separation of two surfaces: (**a**) intermolecular van der Waals (vdW) interactions, (**b**) chemical bonding, (**c**) capillary interactions, (**d**) mechanical interlocking, (**e**) suction forces, (**f**) diffusion of one surface material into the other contacting material, and (**g**) electrostatic and (**h**) magnetic forces. δ+ and δ− (panel (**a**)) illustrate the instantaneous formation of dipoles; *R* (panel (**c**)) indicates the curvature of the meniscus; *p* (panel (**e**)) indicates pressure; and N and S (panel (**h**)) denote the north pole and south pole, respectively （Adapted with permission from Ref. [54]. Copyright 2014, the Annual Reviews）.

The adhesive phenomenon of most creatures originates from contact surfaces. The adhesion mechanisms based on the surface properties include not only van der Waals (vdW) forces, but also chemical bonding, capillary interactions, mechanical interlocking, suction forces, diffusion, electrostatic forces, and magnetic forces [54] (Figure 2).

**Figure 3:**
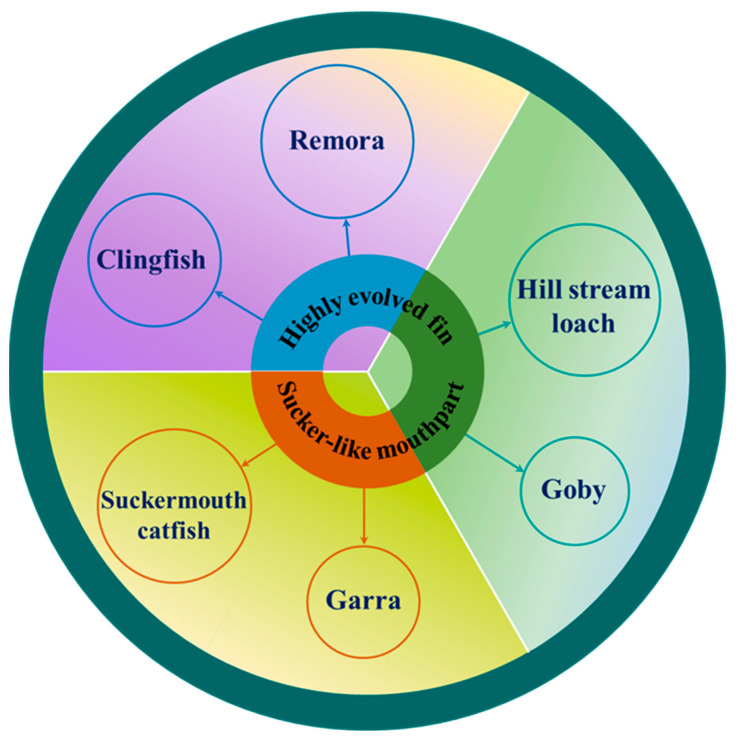
Schematic representation of the classification of fish according to their different adhesion organs.

In fact, adhesion for fish is more complicated. Figure 3 shows the species of each type of adherent fish, which can be divided into three groups depending on the organ of adhesion and the mechanism of adhesion. Among these species of adherent fishes, adhesive structures, movement patterns, swimming speeds, behavioral mechanisms, living environments, weights, and lengths all differ, yielding various strengths and weaknesses. Some fish even make use of multiple adhesion organs and adhesion mechanisms that can be considered when designing underwater adhesion systems. The following subsections will cover the specific characteristics of each species.

### 2.1. The Fin for Adhesion

Underwater adhesion is in high demand in the fields of engineering, materials, chemistry, and even medicine, and fish species with suction discs that evolved from fins are of interest because of their superior performance and interesting structure. At present, although the adhesion mechanisms of terrestrial species have been studied by many scholars, the van der Waals forces and the capillary forces of most terrestrial species are ineffective in underwater environments [55]. Compared with dry adhesion on land, underwater adhesion is more complex and unstable. Thus, the ability of fish such as clingfish and remora to adhere tightly to various objects underwater using highly modified fins has inspired researchers to further investigate stable underwater wet adhesion.

#### 2.1.1. Clingfish

**Figure 4:**
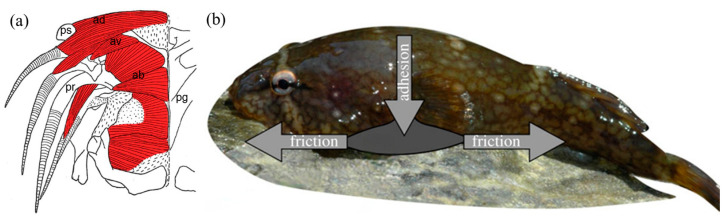
Schematic diagram of the tissue structure of clingfish and the stress on it. (**a**) Bottom view of the tissue structure of the suction disc of the clingfish (Reprinted from Ref. [57,58]). (**b**) Two types of forces act on the clingfish (*Gobiesox maeandricus*) adhesive disc (Reprinted with permission from Ref. [2]. Copyright 2013, the Royal Society). pg, pelvic girdle; ad, arrector dorsalis; av, arrector ventralis; ps, pelvic spine; ab, abductors; pr, pelvic rays.

Clingfish (Gobiesocidae) live in a neritic zone where waves often crash into the coast randomly. To avoid being swept away by a wave, the pelvic fins and part of the pectoral fins of the clingfish merged together to evolve into an adhesive disc. The adhesive disc can help the clingfish fix themselves to rocks, shells, plants, and other fish in the sea. Surprisingly, the adhesion force of the adhesive disc is equivalent to 0.2–0.5 atmospheres below ambient pressure and 80–230 times the weight of the fish [2,56]. Scholars studied the mechanism of adhesion for clingfish by anatomy as early as the 1960s. It was observed that the anterior part of the adhesive disc was supported by two triangular pieces of the pelvic girdle joined by connective tissue. This structure could greatly increase the flexibility of the adhesive disc, which was suitable for complex contact surfaces [57]. When the clingfish started to adhere, the musculature including the arrector dorsalis, arrector ventralis, and abductors unfolded the pelvic spine and pelvic rays (Figure 4a), which could increase the adhesive disc area for adhesion [58]. Two factors contributed to the adhesive disc of clingfish for durable adhesion (Figure 4b): (1) the musculature around the disc created a low-pressure zone that formed an adhesion force that acted vertically to the substrate and (2) the static frictional force prevented the disc from sliding parallel to the substrate [2].

**Figure 5:**
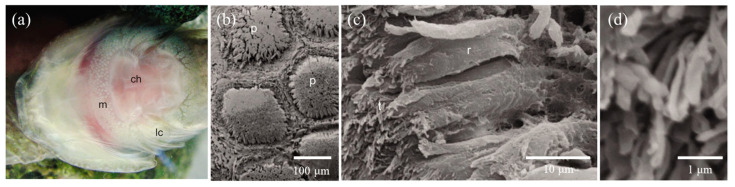
Scanning electron microscopic image of the hierarchical structures on the ventral adhesive disc (Reprinted with permission from Ref. [60]. Copyright 2014, Company of Biologists Ltd.). (**a**) The ventral adhesive disc of the clingfish. (**b**) SEM of the ventral surface of the adhesive disc showing the covering tiled papillae. (**c**) SEM of a papilla, consisting of multiple rods clustered together. (**d**) SEM of the thin filaments on the tip of the rod-like structure. lc, lateral cleft; ch, inner chamber of suction disc; m, disc margin; p, papillae; r, rods; t, tips.

Furthermore, it was worth mentioning that the adhesive disc of clingfish also had a hierarchical structure [3]. The edges of the disc were covered with tiled papillae, as observed with SEM (Figure 5a,b). Further observation revealed that the surface of tiled papillae consisted of many rods with thin filament tips (Figure 5c,d). When clingfish performed static adhesion, these rods generated greater friction and formed an effective seal on rough surfaces for adhesion. Unlike geckos and arthropods, the hierarchical microstructure of clingfish lacked spatulate termini, yet these spatulate termini could increase the flexibility of the microstructure and generate van der Waals forces acting on the substrate [59]. It was suggested that the absence of spatulate structures might be a consequence of the static adhesion for clingfish [2].

#### 2.1.2. Remora

**Figure 6:**
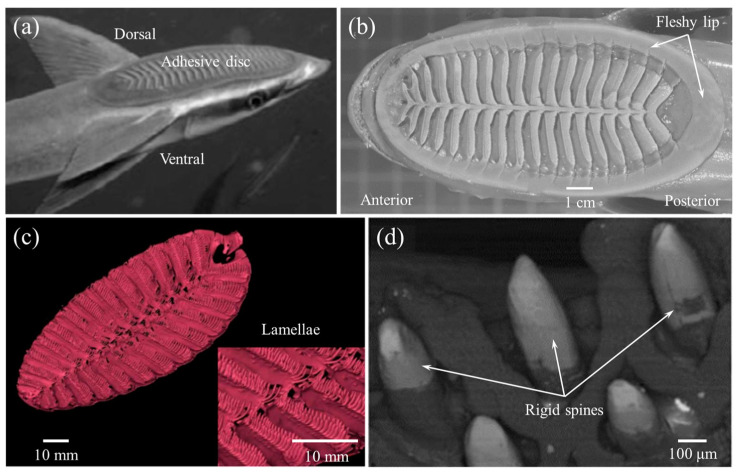
Side view of the remora and the morphology structure of its adhesive disc. (**a**) Side view of a remora (*Echeneis naucrates*) with adhesive disc (Reprinted with permission from Ref. [5]. Copyright 2013, Springer Nature). (**b**) Image of the remora’s adhesive disc showing the soft fleshy lip’s functional features (Reprinted with permission from Ref. [62]. Copyright 2020, John Wiley and Sons). (**c**) A 3D reconstructed model of the remora disc. (Inset) Closer image of the lamellae and rows of spinules (Reprinted with permission from Ref. [33]. Copyright 2017, the American Association for the Advancement of Science). (**d**) SEM micrograph of tapered tips of spinules (Reprinted with permission from Ref. [5]. Copyright 2013, Springer Nature).

Known for their “hitchhike” behavior [33,61], remoras (Echeneidae) live mainly in tropical, subtropical, and temperate seas. As shown in Figure 6a, their heads are flat and their dorsal fins have evolved into adhesive discs that adhere to large marine animals such as sharks, turtles, and cetaceans, or man-made marine equipment like submarines. This behavior can increase the range of movement without swimming and save energy for remoras. The remoras also can accompany their hosts to prey or feed directly on their hosts’ scraps.

In fact, the adhesive disc of remoras can withstand powerful shear forces caused by fluid, especially at high speed for hosts like dolphins [63]. Researchers found that larger forces were required to separate remora from shark skin [64]. Based on histological anatomy and optical image analyses, the amazing adhesion behavior was due to their unique disc structure with three main parts: (1) a soft fleshy lip (Figure 6b) around the edge of the disc, which formed a good seal area for the disc; (2) the lamellae (Figure 6c) arranged at bilaterally symmetrical rows, swung by the erector and depressor muscles inside the disc; and (3) the small rigid and tapered spinules at the edge of lamellae (Figure 6d) that move with lamellae, which could expel air from the disc for seal. Furthermore, the spinules could form an interlocking structure with the microstructures of the body surface of sharks, dolphins, and so on.

Furthermore, researchers conducted tensile tests perpendicular to/along the fiber axis and compression tests along the fiber axis in quasi-static mechanical property tests of remora adhesive discs, respectively. The tests showed that the tensile stress–strain responses of remora adhesive discs displayed non-linear and inelasticity, and the tensile responses along the circumferential and radial directions were very similar. The tensile modulus in the vertical direction is 2–5 times greater than the circumferential and radial modulus, and two orders of magnitude greater than the compressive modulus in the vertical direction, which shows obvious mechanical anisotropy [30]. This mechanical anisotropy is believed to be the result of the combined effect of its unique spinules and collagen fibrous structure [5,30,33,64].

### 2.2. The Mouthpart for Adhesion

Unlike the highly evolved fin that provides strong adhesion forces, fish that use sucker-like mouthparts for attachment generally have limited adhesive strength. Attachment with sucker-like mouthparts tends to be more oriented toward providing a fulcrum for underwater scraping behavior rather than against rapids. In this subsection, we describe two fish that use sucker mouthparts supplemented with microscopic structures for adhesion. The macroscopic and microscopic levels collaborate and complement each other, working to provide new biological inspiration for underwater adhesion.

#### 2.2.1. Suckermouth Catfish

**Figure 7:**
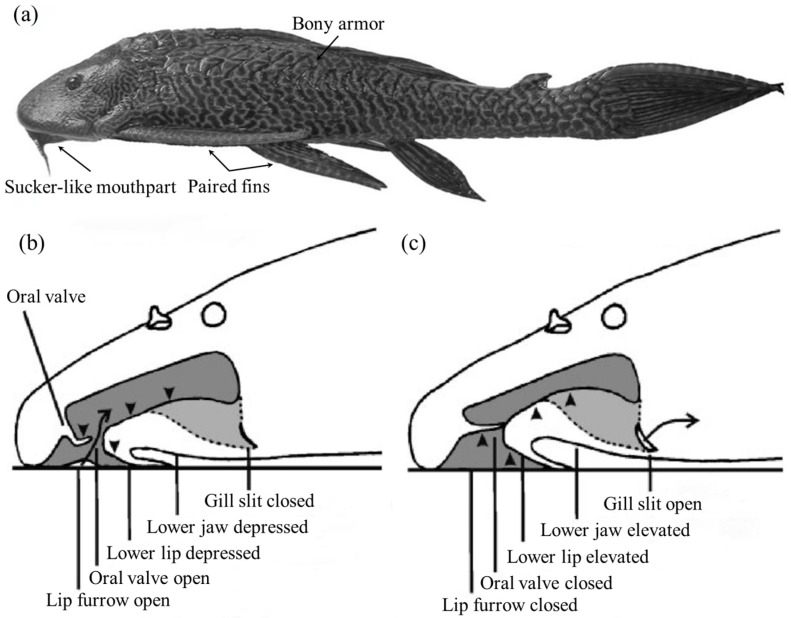
Body structure and the attached respiration cycle of suckermouth catfish. (**a**) Morphological features of the suckermouth catfish: asymmetrical airfoil body shape, bony armor, and ventral sucker-like mouthpart (Reprinted with permission from Ref. [7]. Copyright 2009, University of Chicago Press). (**b**) Near the end of attached inspiration: the lip furrow is open, the post-valvular cavity (larger) is expanded while the pre-valvular cavity is compressed (arrowheads), the lower lip and jaw are depressed, and the gill slits are closed (Reprinted with permission from Ref. [67]. Copyright 2010, John Wiley and Sons). (**c**) Near the end of attached expiration: the lip furrow is closed, the post-valvular cavity is compressed (arrowheads) while the pre-valvular cavity is expanded, the lower lip and jaw are elevated, and the water is expelled through the opened gill slits (Reprinted with permission from Ref. [67]. Copyright 2010, John Wiley and Sons).

The suckermouth catfish (Loricariidae) is a family of benthic fishes that live in Neotropical freshwater areas. The habit of living in fast currents has led the suckermouth catfish to evolve an asymmetrical airfoil body shape (Figure 7a), which is essential for adherent swimming, as this body shape not only helps to reduce resistance to swimming but also increases adhesion [65]. However, it was found that the main adhesion force was generated by the low pressure caused by fast-flowing respiratory streams through narrow passages and channels [65]. The sucker-like mouthparts were used for respiration, eating, and adhesion to the substrate, but respiration did not negatively affect adhesion, and there was a constant sub-ambient pressure in the sucker cavity without leakage-like phenomena. It was shown that the lower lip, lower jaw, and oral valve together controlled the volume changes between the pre-valvular cavity and post-valvular cavity when the suckermouth catfish was respirating (Figure 7b,c). Even during expiration, the suckermouth catfish could make the pre-valvular sucker cavity sealed by closing the lip furrow to assure sucker function, while the water was expelled through the opened gill slits [66,67] (Figure 7c).

**Figure 8:**
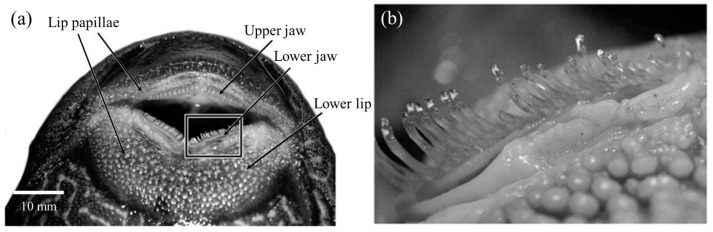
Mouth structure of *Pterygoplichthys disjunctivus*. (**a**) Ventral view of the mouth, showing rows of teeth on the upper and lower jaws and the dermal papillae covering the lip tissue. (**b**) Detail of teeth and dermal papillae (indicated by the box in (**a**)) (Reprinted with permission from Ref. [7]. Copyright 2009, University of Chicago Press).

**Figure 9:**
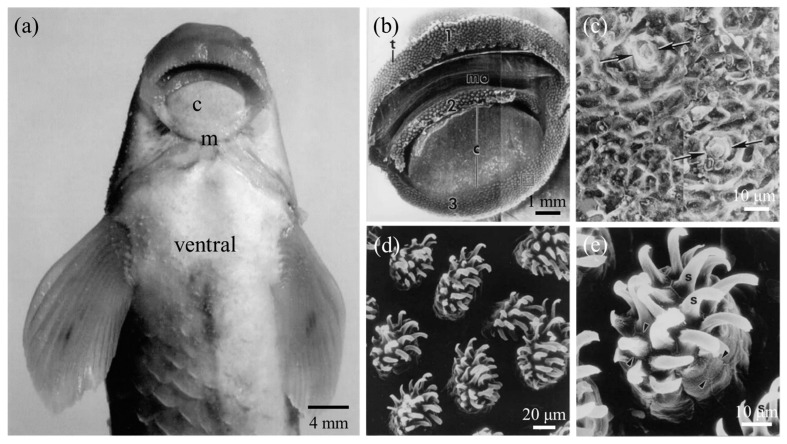
Ventral view of *Garra gotyla gotyla* and the scanning electron microscope image of its sucker. (**a**) Note the central mucogenic region and the marginal keratinized region below the mouth. (**b**) Overview of *Garra*’s sucker, including the central mucogenic region and the marginal keratinized region. (**c**) Mucous pores in the central mucogenic region at high magnification (between the arrows). (**d**) Dense distribution of tubercles in the marginal keratinized region of *Garra*’s sucker. (**e**) A cluster of unculi borne on a single excrescency, and epidermal cells at the base of the spines on a unculi show hexagonal outlines (shown by arrowheads) (Reprinted with permission from Ref. [10]. Copyright 2006, Elsevier Ltd.). c, the central mucogenic region; m, the marginal keratinized region; 1, the upper jaw sheath; 2, the lower jaw sheath; 3, the posterior free border; t, tubercles; mo, mouth; s, spines.

Detailed observation of the mouthparts of suckermouth catfish revealed neatly arranged tooth plates on their upper and lower jaws [7] (Figure 8a). As shown in Figure 9b, these teeth were curved outward relative to the working plane of the sucker-like mouthpart to form a strong mechanical hook-up with the substrate and perform scraping tasks during eating. Additionally, the lower lip was densely covered with various shapes of papillary structures (Figure 8b), which greatly increased the friction with the substrates and provided effective adhesion even on smooth substrates such as glass [7,8].

#### 2.2.2. Garra

The genus *Garra*, a fish of the family Cyprinidae, also uses the disc of the mouth for adhesion. This adhesive disc is behind the arched lower lip and is separated by a crescent-shaped furrow [12] (Figure 9a). *Garra* needs to regulate muscular effort to expel water under the disc to create a vacuum with minimal energy expenditure, thus generating a negative pressure suction proportional to the vacuum [12,68,69]. During this time, the crescent-shaped furrow is used to regulate the pressure gradient [12]. This adhesion ability helps them to inhabit fast-flowing turbulent hill streams and scrape algae and plant debris from rocks, pebbles, and gravel of the streams [70].

In addition, many microscopic structures on the disc of *Garra* were found to play an important role in aiding their firm underwater adhesion. Their adhesive discs were divided into central mucogenic and marginal keratinized regions [68] (Figure 9b). The central mucogenic region was characterized by hexagonal epithelial cells, and these cell boundaries were adjacent to the growing excrescencies with unculi [12,71]. The surface of the epithelial cells had well-developed mucous pores (Figure 9c) and mucosal gland openings [12,70,72], showing a high level of active secretory activity. The secretion of mucus might be related to the frequent frictional and adhesive movement of *Garra*, which could lubricate the surface of the substrate and protect the epithelial cells of the discs from abrasion. The keratinized marginal surface of the disc was densely packed with many tubercles (Figure 9d,e), each modified by a cluster of spine-like unculi [10,72,73,74]. These globular tubercles regulated the pressure gradient of the disc during underwater adhesion, helping the fish to remain firmly anchored to the submerged substrate surface. Also, the sharp unculi were thought to be an adaptation of *Garra* to scrape food from the substrate surface.

### 2.3. Both for Adhesion

The highly evolved fin and sucker-like mouthpart work in conjunction with each other to enable adhesive movement. In this subsection, we present two fish species that use evolved fins and sucker-like mouthparts for adhesive motility, including the hill stream loach and goby, which can escape quickly underwater and even climb rapidly over vertical surfaces. The mechanisms of adhesive movements and the structures of the organs that work with them are more complex, but their study is of substantial importance.

#### 2.3.1. Goby

**Figure 10:**
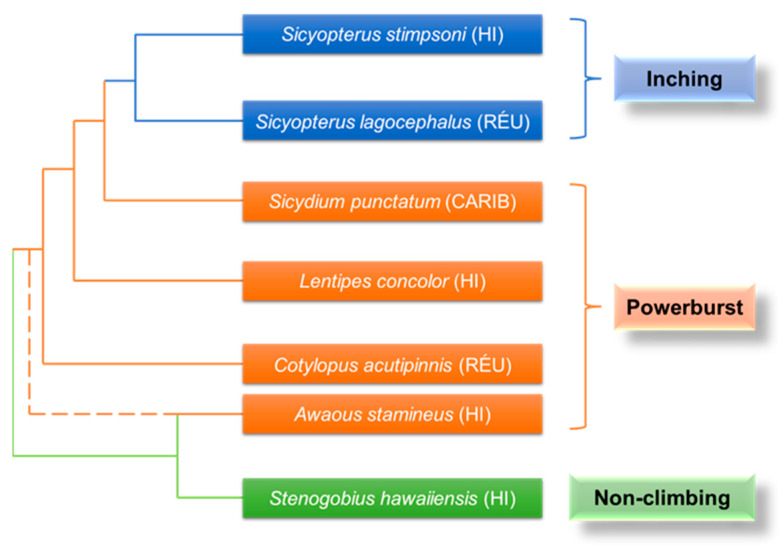
Phylogenetic relationships of the Gobiidae and their classification by climbing mode. The range of each species is shown in parentheses after its name: HI, Hawai’i; R$\overset{´}{E}$U, R$\overset{´}{e}$union; CARIB, Caribbean (Dominica). The non-climbing species *Stenogobius hawaiiensis* is also included. Dashed line indicates the different phylogenetic relationships of the genus *Awaous* (Adapted from Ref. [22]. Adapted with permission from Ref. [75,76,77]. Copyright 2014, Elsevier Ltd., Copyright 2021, John Wiley and Sons, and Copyright 2020, Elsevier Ltd.).

**Figure 11:**
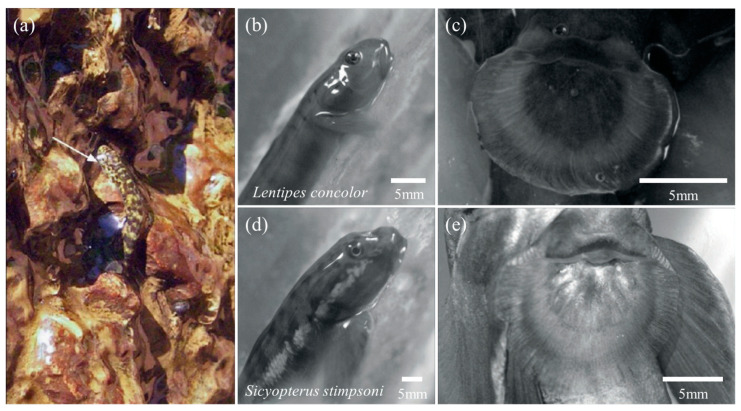
Pictures of gobies climbing a rock face and their lateral and ventral views. (**a**) Photograph of juvenile *Sicyopterus stimpsoni* (arrow) climbing on a vertical rock face (Reprinted with permission from Ref. [78]. Copyright 2008, Elsevier Ltd.). (**b**) Lateral view of *Lentipes concolor*. (**c**) The pelvic sucker of *Lentipes concolor* is formed by the fused pelvic fins. (**d**) Lateral view of *Sicyopterus stimpsoni*. (**e**) Pelvic sucker of *Sicyopterus stimpsoni* (Reprinted with permission from Ref. [20]. Copyright 2012, Company of Biologists Ltd.).

Two different forms of climbing have evolved in gobies (Gobiidae) that climb waterfalls: an extensive powerburst climbing mode that uses fused pelvic fins (pelvic suckers) in conjunction with rapid body wiggling and an inching climbing mode that alternates pelvic suckers with mouthparts shown only in the genus *Sicyopterus* [18,22], which evolved from an ancestor of powerburst climbing (Figure 10). They have evolved an amphibious life cycle, with adults living in freshwater streams and rivers, and juveniles being washed out to the ocean to develop, exhibiting surprising waterfall-climbing behavior as they migrate back to the freshwater habitat of the adults, as shown in Figure 11a. This ability to move with adhesion on near-vertical surfaces allows them to complete migrations of over 10,000 times their body length [18]. 

The *Lentipes concolor* (Figure 11b) and *Awaous guamensis* from Hawai’i are typical powerburst climbers. They were found to start their climb with a single rapid inward retraction of the pectoral fins and sustained subsequent climbs with rapid wiggles of the entire body. This type of climb was rapid (12.4 ± 1.0 BL (body lengths) s^−1^) but short (0.07 ± 0.02 s) [18,19]. During resting, such climbers adhered to the substrate using ventral suckers (Figure 11c) formed by the fused pelvic fins [79]. Furthermore, the posterior edge of their pectoral fins had substantial contact with the substrate before climbing and was used to provide an initial acceleration from a stationary start. However, sequential cycles of pectoral fin adduction appeared to be used only for minor positional adjustments on the vertical substrate, rather than for driving long climbing cycles [18,79,80,81,82]. It was thought that this might represent a new movement mode: aquatic propulsion supplements the mechanisms for the generation of terrestrial thrust [76]. In contrast, inching climbers, represented by *Sicyopterus stimpsoni* (Figure 11d), relied on the alternate attachment of mouthparts and pelvic fin suckers (Figure 11e) to achieve inch-by-inch climbing on vertical substrates [21,83,84,85]. Their juveniles develop in the ocean for up to six months and undergo a rapid metamorphosis (48 h) before climbing, including an enlargement of the upper lip, a shift in mouth position from terminal to ventral, and a sudden loss of weight [18,84]. This allows them to climb inch-by-inch for several seconds at a rate of 0.21 ± 0.01 BL s^−1^: the mouthpart first attaches to the substrate, the posterior body is pulled up, and with the attachment of the pelvic sucker, the mouthpart releases and the anterior body continues to advance [18]. It was found that the suction force of the mouthpart was much lower than that of the pelvic sucker [20,85,86]. This also confirmed that the pectoral fins spread maximally over the climbing surface during mouthpart attachment, aiding crawling by imparting maximum contact and friction [20].

#### 2.3.2. Hill Stream Loach

**Figure 12:**
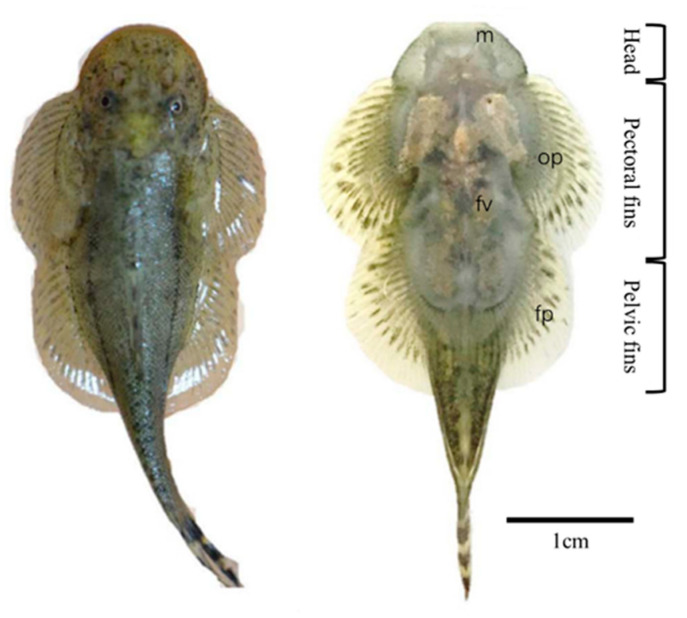
Dorsal and ventral views of a hill stream loach (*Sinogastromyzon puliensis*). This fish has an adhesive system consisting of a mouthpart, overlapped pectoral fins, and fused pelvic fins. m, mouthpart; op, overlapped pectoral fins; fv, flat ventral side; fp, fused pelvic fins (Reprinted with permission from Ref. [17]. Copyright 2017, Elsevier Ltd.).

The hill stream loach (Balitoridae) can use its whole body as a suction system to cope with the living environment of rapid water flow. This suction system mainly consists of the mouthpart, overlapped pectoral, and fused pelvic fins (Figure 12). The surface areas of the pectoral and pelvic fins are enlarged by the increased number of fin rays, thus enhancing the suction and friction force [87]. What is more, the hill stream loach has a rather well-developed fan-like protractor ischii. The protractor ischii pulls the pelvic girdle anterodorsally, creating a small cavity between the ventral pelvic surface and the substrate [87,88], which, together with the effective attachment of the fins, results in a negative pressure underneath the body.

The overall flattened shape of the fish’s body reduces the resistance caused by the impact of the water. Only the head profile abruptly rises, supported by the enlarged lacrimal bone, along with the posterior fin rays held upward, creating a strong downward hydrodynamic force: when facing the current, the body is pressed on the base [87,88]. The flat ventral is held close to the substrate, preventing upward hydrodynamic forces from being generated. When the hill stream loach moves adherently, it uses two suckers: one comprising the pectoral fins and mouthpart and the other comprising the pelvic fins. The two suckers undulate left–right: one side of the former lifts to separate from the substrate and move forward, while the latter remains attached. When the pectoral fin is reattached to the substrate, the latter sucker is carried forward as the other side of the body is raised. The crawling-like movements of the body and the paired fins, with the caudal fins playing only a supporting role, aid this unique form of locomotion [87,89]. As they move forward in this manner, part of the body remains attached to the substrate and the inflow and outflow of water under the body is effectively controlled, achieving a good adhesive movement.

**Figure 13:**
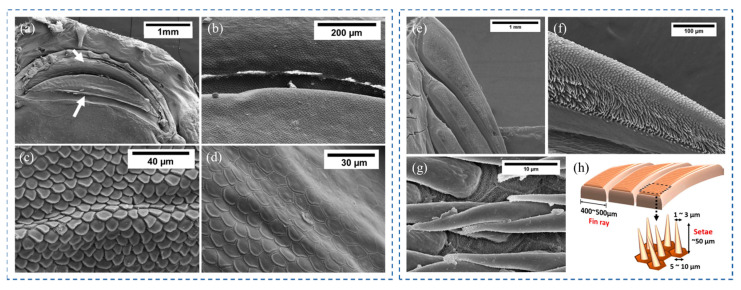
SEM images of the mouth and fin ray of a hill stream loach. (**a**) The white arrows point to the upper and lower lips, respectively. (**b**) Top view of the lip under higher magnification. (**c**) Thick unculi near the inner mouth. (**d**) Thin unculi near the outer mouth. (**e**) The fin rays are densely covered with setae. (**f**) Setae worn off the outer edge of the fin ray. (**g**) The hexagonal boundary of the cells indicates that these setae are keratinized epithelial cells. (**h**) The hierarchical structure of the fin ray surface (Reprinted with permission from Ref. [17]. Copyright 2017, Elsevier Ltd.).

As for the microscopic level, microscopic structures that facilitate adhesion were also found to be present in hill stream loaches. As shown in Figure 13a,b, the surface morphology of the upper and lower lips of the hill stream loach was covered with polygonal pad-like protrusions, called unculi. Compared with the unculi near the outer mouth (Figure 13c), the unculi at the inner mouth were thicker, and the grooves between the unculi were deeper [17] (Figure 13d). The main function of the unculi seems to be to achieve a sealing sucker effect by entrapping micro bubbles [16]. It has also been suggested that the unculi can be used as hooks to interlock with vegetative or irregular structures on the substrate [15,17]. In conclusion, these keratinized epithelium cells, which resemble the scales of a snake, are thought to play a crucial role in generating negative pressure and friction in hill stream loaches. Furthermore, it was found that the fins of the hill stream loach consisted of parallel-arranged fin rays. The fin rays were densely covered with setae (Figure 13e), and the outermost edge of the setae was worn off and became flat (Figure 13f). The setae were elongated conical in shape, about 30~50 μm in length, 1~3 μm in diameter at the tip, and 5~10 μm at the root [17] (Figure 13g,h). The root of each seta was attached to a hexagonal epithelial cell. This fin ray–setae hierarchical structure was thought to adapt to surfaces of different roughness, thereby improving adhesion.

There are also many fishes that can rely on evolved adhesion organs such as mouthparts or fins to adhere underwater and even climb through wading. Consider *Psilorhynchus nepalensis* and *Myersglanis blythii*, which achieve underwater adhesion utilizing flexible hooks on the ventral fin pads of their pectoral or pelvic fins [90]. Or consider *Entosphenus tridentatus*, a migratory climber on vertical surfaces, which uses a burst-and-attach mode of movement to provide adequate adhesive surfaces [91]. Furthermore, *Kryptolebias marmoratus* and *Cryptotora thamicola* use ventral adhesion mechanisms to transition from water to land by launch or paired fin assistance, which is thought to represent a new form of terrestrial transition for non-tetrapodal [92,93]. The behavior of fish by adherence with the use of paired fins is also believed to be related to the origin of the limbs in the tetrapod lineages [94,95,96,97,98]. But these are not all the species, just a few samples of them.

## 3. Underwater Adhesion Systems

**Figure 14:**
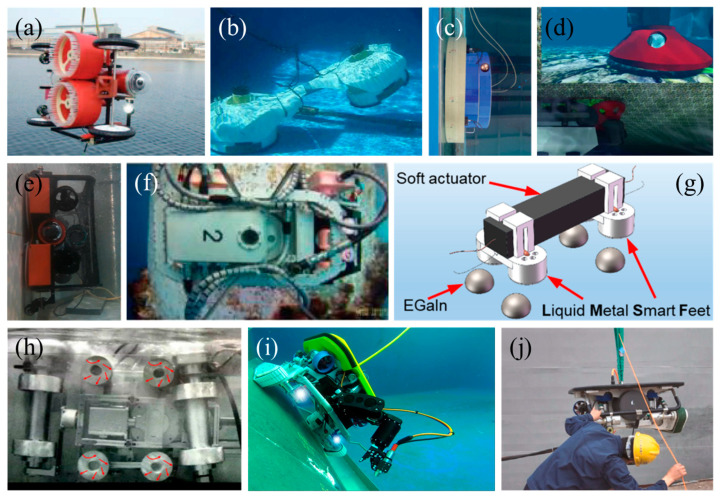
Non-bioinspired underwater adhesion systems. (**a**) Hull cleaning robot with adsorption generated by two propellers (Reprinted with permission from Ref. [99]. Copyright 2012, IEEE). (**b**) Unconventional underwater robot for cleaning complex hulls (Reprinted with permission from Ref. [100]. Copyright 2013, IEEE). (**c**) Underwater crawling adsorption robot (Reprinted with permission from Ref. [101,102,103]. Copyright 2021, AIP Publishing). (**d**) Multi-functional tugboat for monitoring and cleaning bottom fouling of ships (Reprinted from Ref. [104]). (**e**) Underwater dual-mode operating robot with propeller-driven Bernoulli adsorption device (Reprinted with permission from Ref. [105]. Copyright 2022, Elsevier Ltd.). (**f**) Variable diameter robot for cleaning underwater steel pipes (Reprinted with permission from Ref. [106]. Copyright 2018, Emerald Group Publishing Limited). (**g**) Underwater crawling robot with liquid metal smart feet (Reprinted with permission from Ref. [107]. Copyright 2021, American Chemical Society). (**h**) Underwater climbing robot using the principle of negative pressure (red arrows indicate the direction of the flows) (Reprinted from Ref. [108]). (**i**) ROVING BAT (Reprinted from Ref. [109,110]). (**j**) Underwater cleaning robot driven with six thrusters and two crawler belts (Reprinted with permission from Ref. [111]. Copyright 2023, Elsevier Ltd.).

Most non-bioinspired underwater adhesion systems are used primarily for underwater hull cleaning, inspection, and maintenance. These cleanings include biological contamination, such as algae, barnacles, and mussels, which studies have shown not only affects the normal speed of a vessel but also leads to consuming more fuel and emitting more exhaust fumes. Hull inspections and maintenance include rudder and propeller inspections, cathodic protection, and splash zone inspections, which significantly reduce the potential navigational risk. The main types of attachment for non-bioinspired underwater adhesion systems are propeller-generated adsorption, negative pressure adsorption, magnetic adsorption, and thruster reaction force attachment. Figure 14 shows some examples of non-bioinspired underwater adhesion systems in recent years.

**Figure 15:**
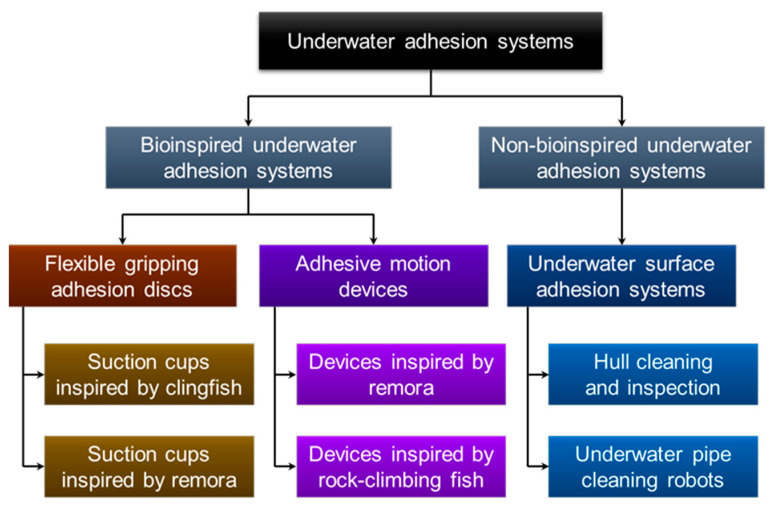
Classification of bioinspired and non-bioinspired underwater adhesion systems.

Compared with dry adhesion, underwater adhesion has challenges such as low strength, instability, and difficulty in regulation. It is clear that these non-bioinspired underwater adhesion systems are deficient in terms of adhesion strength, adhesive movement, and overcoming obstacles. Bio-inspiration provides an innovation path for underwater operational missions. Researchers learn from nature and replicate adhesion mechanisms, unique microstructures, and the adaptive morphology of adherent fish for use in underwater systems such as flexible underwater grasping devices, benthic underwater vehicles, and hull cleaning robots, thus solving the problem of underwater adhesion with the added benefit of protecting the marine environment and resources. As shown in Figure 15, there are two subcategories within the bioinspired category: flexible gripping adhesive discs and adhesive motion devices.

In recent years, flexible polymer-based adhesive discs have been widely used in complex working conditions such as underwater flexible gripping. In this section, we categorize bionic adhesive discs inspired by adherent fish into two groups: (1) bionic adhesive discs that can accomplish underwater flexible gripping and (2) underwater adhesive motion devices with actuators. Table 1 and Table 2 express each bioinspired underwater adhesion system’s characteristics and pictures.

### 3.1. Flexible Gripping Adhesive Discs

Inspired by the clingfish, a variety of flexible gripping adhesive discs with different materials have been developed. Table 1 summarizes the recent research on the practical use of bionic clingfish discs. Ditsche et al. [27] developed a biological suction disc with a multi-material layered structure. The stiff silicon material (Young’s modulus: 8 MPa) resembles harder bones in the clingfish suction disc, while the highly elastic silicon (Young’s modulus: 0.2 MPa) resembles the soft disc rim, which is capable of attaching to surfaces with a roughness of 270 µm grain size with a tension of up to 70 kPa. Friction measurements indicate that the increased friction of the suction disc edges on rough substrates contributes to improved tenacity, delayed failure, and increased adhesion. Sandoval et al. [28] were also inspired by the clingfish adhesion mechanism and designed an artificial suction disc using contact visualization techniques and Finite Element Analysis to relate the effects of the clingfish’s disc gap, soft outer layer, and body geometry to adhesion performance (Table 1). The suction discs are made from silicone elastomer spin-coated 3D printed mold, weighing approximately 2 g, and are capable of gripping concave surfaces with a small radius of curvature (12.5 mm), support payloads of up to 0.7 kg, and achieve adhesion of 14.3 ± 1.5 kPa on submerged rough surfaces (grain size, 269 μm) [28].

The dorsal sucker of the remora is also an important bionic model for researchers. In 2019, Lee et al. [29] used 3D printing and soft lithography to fabricate a silicon-based elastomeric adhesive modeled on the dorsal suction discs of remoras (Table 1). The adhesive exhibits admirable adhesion properties to glass substrates in water, and the high adhesion and flexibility of the edge lip allow it to maintain airtight conditions and thus isolate itself from external water after the water has been drained from its interior. The adhesive has a pull-off strength of 26.68 N cm^−2^ (266.8 kPa) and a shear strength of 19.42 N cm^−2^ (194.2 kPa), which shows stable adhesion not only on smooth surfaces but also on rough surfaces [29]. In addition, durability tests show that the adhesion and friction properties remain good after several uses. In 2020, Su et al. [30] achieved anisotropic mechanical and enhanced adhesion properties of a soft underwater bionic adhesive by analyzing the fibrous structure of the unique vertically oriented collagen fibers of the remora suction disc and embedding the vertically oriented nylon fibers into the soft silicone matrix using electrostatic flocking (Table 1). In this regard, the soft silicone matrix ensures adequate contact and the millimeter-long fibers implanted along the vertical direction ensure firm adhesion, thus forming a composite with a vertical tensile modulus of 1000 kPa and a compressive modulus of 70 kPa. This is in line with the high tensile and low compressive modulus shown by the 3D fibrous network in the suction discs of the remora fish and the vertical collagen fibers in its central section. In experiments with silicone controls, the bionic adhesive showed a maximum increase in pull-off force of 62.5% and an increase in adhesion time of 340% [30].

Both in terms of adhesion strength and adhesion time, the underwater bionic flexible gripper inspired by adherent fish outperforms commercial suction cups underwater. In particular, for underwater anisotropic media surfaces and rough media surfaces, the bionic adhesive discs far outperform commercial suction cups. This also highlights their potential for applications in complex and fragile marine environments as well as in medical applications. However, limitations in processing accuracy and material properties leave a performance gap between bionic adhesive discs and real adherent fish, and next steps need to be taken, for example, the development of synthetic muscles or skin to achieve more reliable underwater adhesion.

### 3.2. Adhesive Motion Devices

Based on detailed morphological and kinematic investigations of remora, a variety of vehicles or robots have been developed that are capable of adhesive motion underwater. In 2017, Wen et al. [33] designed an underwater vehicle with a multi-material bionic adhesive disc that is capable of automatic adhesion to and detachment from smooth, rough, and compliant surfaces underwater (Table 2). They used multi-material 3D printing to create a disc structure with a stiffness spanning three orders of magnitude and laser processing to create carbon fiber spinules (270 μm base diameter) attached to a soft actuator-controlled lamellae structure. As the lamellae structure rotates, the carbon fiber spinules and soft material engage with the surface to create a pulling force of 340 times the weight of the adhesive disc [33]. The underwater vehicle equipped with a bionic adhesive disc allows remote control and flexible switching between the swim and attachment modes. As shown in Table 2, the team then developed a remora robot, propelled by a jet of water, to study the detachment mechanisms of remora suckers [34]. The robot mimics the three stages of remora fish disengagement behavior, namely, (1) folding down lamellae is essential to reduce the disengagement resistance of the sucker (vertical interface forces and friction forces), and rolling up the soft lip can also break the adhesive seal and reduce vertical pull-off force up to 94 times; (2) the partially elastic base of the sucker (Young’s modulus: ∼3 MPa) can save 30% of the power consumption compared with a rigid base (Young’s modulus: ∼3 GPa); and (3) as the lamellae fold and the entire soft lip uncoils, the corresponding drag wake flow is reduced by 44% compared with the connected state [34]. Underwater robots with integrated remora-inspired suckers have hitchhiking and pick-and-place capabilities, which provide the basis for the development of untethered, multimodal underwater hitchhiking robots.

In addition to simple adhesion and detachment movements, the team studied the adhesive gliding behavior of remora and designed a bionic robot that can switch freely between zero, low friction, and strong adhesion states [35]. The biomimetic suction disc controls the disc lip and lamellar movement under actuation, utilizing soft actuators that can “compression-rotation” and “compression-extension” with just one degree of freedom. The bionic suction disc with a low-modulus soft lip can adhere to smooth submerged surfaces with a preload of 0.1N and can control normal adhesion and tangential friction from ~10^−1^ to ~10^2^ N and from ~10^−1^ to ~10^1^ N. Mounted on an underwater robot with a bionic pectoral fin flapping assisted swim, it enables underwater attachment, detachment, skimming, and sliding movements [35]. Following this, Wen et al. [36] created an aerial–aquatic hitchhiking robot in 2022, thus greatly increasing the robot’s range of movement (Table 2). The robot can cross the air–water boundary in 0.35 s. In addition to this, the robot can rapidly attach and detach on challenging surfaces such as curved, rough, moving, incomplete, and biofouling surfaces in the air and underwater with minimal oscillation for long-duration adhesion. This provides important support for future robots for autonomous biological detection, monitoring, and tracking in a variety of aerial–aquatic environments.

Furthermore, inspired by the movement of the hill stream loach (*Beaufortia kweichowensis*), in 2022, Wang et al. [37] developed a bionic robot that adheres to underwater surfaces to achieve forward and backward crawling (Table 2). The robot consists of two anisotropic adhesive components and a linear actuator, each anisotropic adhesive component comprising a commercial sucker and two retractable bioinspired fin components. The fin components mimic the abduction and adduction of the pectoral and pelvic fins of the hill stream loach with retractable sections to enable surface movement without detaching from the substrate [37].

Inspired by the mechanisms of adhesion, detachment, and sliding along surfaces of various adherent fish, underwater bionic adherent locomotion systems with adhesion, detachment, skimming, and sliding capabilities have been developed for a wide range of complex aquatic environments. At the same time, underwater adhesion will increasingly be used in research areas such as cross-medium vehicles or robotics to support complex systems in multi-functional, multi-modal, and multi-scenario situations. This demonstrates the importance of using bioinspired adhesion principles in underwater adhesion applications. The development of this technology will not only involve the maritime, military, and energy sectors where humans are present, but will also have a profound impact on ecosystem conservation, biodiversity conservation, and the protection of natural resources.

## 4. Constraints, Limitations, and Future Recommendations

Section 2 and Section 3, respectively, discuss the gathered characteristics of adherent fishes and bioinspired, non-bioinspired underwater adhesion systems. Through the organization of these fishes and systems, it is clear that bioinspiration, especially that of adherent fish, is important for the development of underwater adhesion systems. Their impact can be seen not only in terms of better underwater adhesion strength, longer underwater adhesion times, and smoother underwater adhesion gliding but also in the ability to perform more difficult underwater tasks, adapt to more complex underwater environments, and produce less underwater ecological impact. However, there are still many constraints and limitations to the existing bioinspired underwater adhesion systems. By comparing bioinspired underwater adhesion systems with biological prototypes, the similarities, differences, and gaps between the two can be better understood. Suggestions will be expressed that will help underwater adhesion systems work toward biomimicry.

With respect to adhesive discs for firm gripping, biological prototypes are mostly based on clingfish and remora fish. Most of the flexible bionic discs based on their suction discs are made of elastic polymers or hydrogel material with processing methods such as 3D printing, photolithography, and etching [112]. The micro/nanostructures on the surface of the suction discs are limited by the manufacturing process and the precision of the fabrication, but these micro/nanostructures are precisely the key to the superior adhesion of the clingfish on rough underwater surfaces. Therefore, developing and manufacturing micro/nano-scale processing equipment will greatly enhance the operational capability and use of underwater adhesion devices. Similarly, existing fish adsorption and friction testing devices are unable to simulate underwater adhesion environments, and in order to further unravel the potential adhesion mechanisms of adherent fish, research on high-precision measuring instruments that are closer to fish survival environments is needed [17,60,82,113]. As bioinspired underwater adhesion devices become more widely used in various fields and specific situations, there is an urgent need to develop new materials and manufacturing methods with responsiveness, programmable, and even anisotropic properties. Biomimetic 4D printing, for example, enables morphological changes in response to external stimuli such as temperature, light, and force and is a promising direction for the design and manufacture of subsequent bioinspired underwater adhesion systems [114,115,116,117,118,119].

For bioinspired adhesion motion devices, adhesion motion is mostly achieved by underwater robots or vehicles carrying detachable bionic adhesive discs, which requires that the bionic discs be highly versatile and capable of loading and unloading. The performance of bionic adhesive discs for adhesive movements on more challenging slurry or solid–liquid mixed media, which is a medium unique to the complex and variable underwater environment, has not yet been studied and proven. In terms of biological prototypes, an adherent fish often has multiple organs that facilitate adhesive movement, or a single biological structure may have multiple functions. Knowing which organs or structures contribute to adhesive movements and how they are coordinated is a challenge for the development of underwater adhesive devices. In addition, an in-depth study of how the adhesive movement of fish increases friction and resists shear, and how adhesive movement, detachment, and sliding behavior is achieved on this basis, requires more specialized systems to test underwater contact behavior. It can also be used for fluid force visualization and mechanical modeling using digital particle image velocimetry systems or computational hydrodynamics [120,121,122,123,124,125,126,127,128,129,130]. With the development of unsteady flow fields and surface attachment physics, the modeling of kinematic and kinetic models for adherent fish, and the refinement of interfacial force theory at the liquid–solid interface, further progress in underwater adhesion kinematics will be achieved [131,132].

In summary, in this section, the limitations and future recommendations for both flexible underwater gripping adhesive discs based on firmly adherent fish and underwater adhesive motion devices based on adherent-motion fish were analyzed. It is important to note that many improved or even disruptive technologies are necessary for stable adhesion and efficient movement on solid–liquid media surfaces in the development of future underwater systems, such as lighter but stronger adhesion mechanisms, versatile operational actuators, and more stable and reliable materials [133]. Finally, taking more inspiration from biology while developing underwater adhesion systems, by integrating all the system components into homogeneous entities, such as hydrogel robots with autonomous and intelligent behaviors, may help with the development of more biological-like systems, thus facilitating the rapid development of marine science and technology [134,135].

## 5. Conclusions

Through natural selection, adherent fish have evolved optimally adhesive structures and/or developed unique adhesive mechanisms to improve their chances of survival in complex underwater environments. A review and analysis of the behavior and characteristics of adherent fish required for the design of bioinspired underwater attachment systems is presented in this paper. A taxonomic and comparative approach was used to facilitate the generalization of the bionic inspiration provided by each adherent fish, which will help other researchers in the field of bioinspired underwater adhesion systems find biological attributes that match their interests in the underwater environment and use this information in the design of underwater systems [136].

Research on bionic adhesion systems is in its infancy, with previous bionic adhesion studies mostly modeled on terrestrial organisms or common aquatic organisms such as octopus and abalone species that have adhesion capabilities. Although adhesion systems inspired by these organisms can solve many adhesion problems in human production and life, they are limited for use in some specific application situations or more complex and harsh environments. For example, octopus-inspired adhesion systems require additional pumping systems and power sources during the adhesion process. The underwater performance of bionic robots modeled on geckos using van der Waals (vdW) forces to adhere to various wall surfaces is unknown. In addition, many adhesion devices are mostly permanently adherent and lack reversible adhesion capabilities. Therefore, more work and more open minds are needed in this field, and bionic applications inspired by adherent fish are expected to provide the special features and advanced performance required.

The field of underwater adhesion and bioinspired underwater adhesion systems research is beginning to take off. Fish use a wide range of adhesion and locomotion techniques, and although they have much in common with each other, they can still be distinguished by their body morphology and micro/nanostructures. These commonalities and characteristics that have evolved between fish can provide key principles for the design of versatile, reliable, and low-energy consumption underwater systems. Accordingly, this review also provides current fish-bioinspired and non-bioinspired underwater adhesion systems, presenting what they can currently achieve and what limitations they still have. Bioinspired systems are discussed in more detail and compared with the corresponding biological prototypes. For example, the micro/nanostructures on the flexible gripping adhesive discs inspired by clingfish and remora are still vastly different from those of biological models due to the limitations of fabrication precision. The adhesive motion devices inspired by the remora and hill stream loach do not have the flexibility and stability of the biological models. These systems are not yet able to perfectly replicate the many details and capabilities of fish adhesion, but with rapid advances in mechanical design, engineering control, high-performance bionic materials, micro/nano processing, and artificial intelligence, more fish-bioinspired adhesion systems for a wide range of applications in the complex and changing underwater world will emerge. In conclusion, fish-bioinspired adhesion systems should be deeply integrated with various application scenarios to play a greater role in underwater missions related to human activities.

## Figures and Tables

**Table 1:** Characteristics and pictures of flexible gripping adhesive discs.

Biological Prototype	Characteristics	Pictures
Clingfish	The suction disc features a multi-material (stiff silicon and highly elastic silicon) layered structure; The suction disc can produce tension up to 70 kPa on the surface with a roughness of 270 μm grain size.	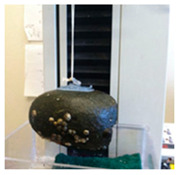 (Reprinted with permission from Ref. [27]. Copyright 2019, the Royal Society)
	The suction disc is made from silicone an elastomer spin-coated 3D printed mold and weighs approximately 2 g; The suction disc can grip concave surfaces with a small radius of curvature (12.5 mm) and support payloads of up to 0.7 kg; The suction disc can provide 14.3 ± 1.5 kPa adhesion to underwater rough surfaces (grain size, 269 μm).	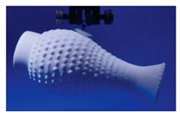 (Reprinted with permission from Ref. [28,32]. Copyright 2019, IOP Publishing)
	The suction cup is made by overmolding silicone onto a commercial suction cup; Regardless of surface texture, this suction cup has a higher stress and working capacity on compliant substrates than the hard commercial cup.	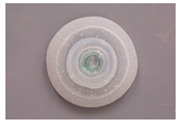 (Reprinted with permission from Ref. [31]. Copyright 2022, Company of Biologists Ltd.)
Remora	The adhesive disc is manufactured with 3D printing and soft lithography processing of silicon-based elastomer; The adhesive disc has a pull-off strength of 26.68 N cm^−2^ (266.8 kPa) and a shear strength of 19.42 N cm^−2^ (194.2 kPa).	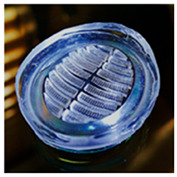 (Reprinted with permission from Ref. [29]. Copyright 2019, American Chemical Society)
	The adhesive disc is made by electrostatic flocking of vertically oriented nylon fibers into a soft silicone matrix; Compared with commercial suction cups, this adhesive disc offers a 62.5% increase in maximum pull-off force and a 340% increase in adhesion time.	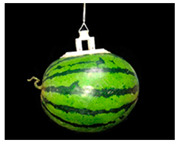 (Reprinted with permission from Ref. [30]. Copyright 2020, Elsevier Ltd.)

**Table 2:** Characteristics and pictures of adhesive motion devices.

Biological Prototype	Characteristics	Pictures
Remora	The bionic sticky disc mounted on an underwater vehicle can automatically adhere to and detach from smooth, rough, and compliant surfaces underwater; Soft actuators control the pulling up of carbon fiber spinules on the lamellae structure to produce 340 times the weight of the adhesive disc.	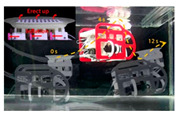 (Reprinted with permission from Ref. [33]. Copyright 2017, the American Association for the Advancement of Science)
	The suction disc folds down the lamellae and rolls up the soft lip during disengagement, resulting in a 94-fold reduction in disengagement resistance; Compared with a rigid base, the partially elastic base of the suction disc saves 30% of power consumption when detached from the substrate.	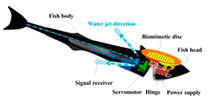 (Reprinted with permission from Ref. [34]. Copyright 2020, IOP Publishing)
	The suction disc can be freely switched between zero, low friction, and strong adhesion states; The bionic suction disc with a low-modulus soft lip can slide over smooth underwater surfaces with a preload of 0.1 N.	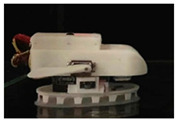 (Reprinted with permission from Ref. [35]. Copyright 2022, IOP Publishing)
	The bionic suction disc is mounted on a cross-medium vehicle and can cross the air–water boundary in 0.35 s; The suction disc can quickly attach to and detach from any challenging surface in the air or underwater.	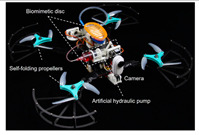 (Reprinted with permission from Ref. [36]. Copyright 2022, the American Association for the Advancement of Science)
Hill stream loach	The robot consists of two anisotropic adhesive components and a linear actuator; The adhesive components have retractable sections to enable surface movement without detaching from the substrate.	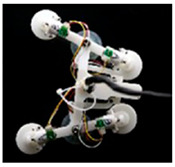 (Reprinted with permission from Ref. [37]. Copyright 2022, Springer Nature)

## Data Availability

Data sharing is not applicable to this article as no new data were created or analyzed in this study.
